# Association of Paraoxonase-1 Q192R (rs662) Single Nucleotide Variation with Cardiovascular Risk in Coffee Harvesters of Central Colombia

**DOI:** 10.1155/2017/6913106

**Published:** 2017-12-21

**Authors:** Fernando Siller-López, Sandra Garzón-Castaño, Martha E. Ramos-Márquez, Iván Hernández-Cañaveral

**Affiliations:** ^1^Grupo de Investigación en Enfermedades Infecciosas, Universidad Católica de Manizales, Carrera 23, No. 60-63, Manizales, Caldas, Colombia; ^2^Grupo de Investigación en Biomedicina, Fundación Universitaria Autónoma de las Américas, Avenida de las Américas, No. 98-56, Pereira, Risaralda, Colombia; ^3^Departamento de Biología Molecular y Genómica, Centro Universitario de Ciencias de la Salud, Universidad de Guadalajara, Sierra Mojada 950, Col. Independencia, 44340 Guadalajara, JAL, Mexico; ^4^Departamento de Microbiología y Patología, Centro Universitario de Ciencias de la Salud, Universidad de Guadalajara, Sierra Mojada 950, Col. Independencia, 44340 Guadalajara, JAL, Mexico

## Abstract

Paraoxonase 1 (PON1), a high-density lipoprotein-associated antioxidant enzyme, hydrolyzes several organophosphate pesticides and oxidized lipids. The PON1 Q192R polymorphism affects the catalytic efficiency and is considered a risk factor for pesticide intoxication and cardiovascular disease (CVD) but the association is not consistent between individuals or populations. We aimed to study the association of PON1 Q192R polymorphism with CVD risk in coffee harvesters of central Colombia. Demographics were collected from 205 subjects via standardized questionnaires. Lipid profiles and serum butyrylcholinesterase (BChE) were measured by standard procedures. The calculated 10-year atherosclerotic CVD (ASCVD) risk was used as the cardiovascular risk estimate. Q192R genotype was determined by real-time PCR. Prevalence of hypertension, hypercholesterolemia, and the 10-year ASCVD risk was 33%, 62%, and 22%, respectively. BChE levels were no indicative of recent pesticide exposure, although a positive correlation was observed with BChE and hypercholesterolemia. The Q192R genotype frequencies were 38% (QQ), 44% (QR), and 18% (RR). We found an association of the 192Q genotype with hypertension. The results of this study signal the importance to evaluate the influence and potential interactions of BChE and PON1 192Q allele with known genetic and environmental factors implicated in the pathogenesis of CVD.

## 1. Introduction

Coffee production is an intensive economic activity in central Colombia. Agricultural workers are usually at high risk of disease due to a frequent exposure to organophosphate pesticides (OPs) and unfavorable conditions like common rural poverty and unbalanced nutritional requirements. OP represented 40% of the pesticides exposed to farming workers and subjects living in areas close to agricultural zones in Colombia [1]. OPs are responsible for several adverse effects on human health. The main mechanism of OP toxicity is the inhibition of acetylcholinesterase (AChE) activity in the central and peripheral nervous system. Exposure to OP is biomonitored by blood analysis of cholinesterase (ChE) enzymatic activity of erythrocyte AChE or serum ChE [butyrylcholinesterase (BChE)] that act as surrogate for neuronal AChE activity and are valid markers of OP biological effects [2]. Acute and chronic exposure to OP is the cause of several types of toxicity, mainly neurotoxicity, but is also associated with hepatic, pulmonary, renal, and cardiovascular alterations [3–5].

In Colombia, cardiovascular disease (CVD) is the first cause of mortality among the general population and four of the departments that represent the Coffee Belt region of central Colombia (Tolima, Caldas, Quindío, and Risaralda) are among the first five positions of the list of CVD mortality by region [6]. Known health behaviors (smoking tobacco use, physical inactivity, suboptimal diet quality, and obesity) and health factors (familial history and genetics, hypercholesterolemia, hypertension, diabetes mellitus, and metabolic syndrome) define cardiovascular health [7]. An enhanced risk to CVD may result when two or more conditions are combined or there is a simultaneous exposure to environmental adverse elements.

Several key biomolecules have long been associated with protection against CVD. HDL (high-density lipoprotein) and HDL-associated enzymes, like the lipolactonase paraoxonase 1 (PON1), have antiatherogenic properties by preventing and reversing LDL (low density lipoprotein) oxidation. PON1 protects LDL from oxidation by the hydrolysis of biologically active lipoperoxides [8] and is also capable of hydrolyzing toxic compounds like certain OP [9, 10]. However, the protective function of PON1 can be modified by genetic regulation that influences the protein level and activity. The gene coding for PON1 has two amino acid polymorphisms that modify the function of the protein and vary widely among individuals and populations, the M55L gene variant (rs854560) that exchanges a methionine for leucine at position 55 (A for T nucleotide change) and the widely studied Q192R gene variant, namely, rs662 (c.575A>G or p.Gln192Arg) that exchanges an arginine (R) for glutamine (Q) at position 192 of the protein (G for A nucleotide change). Results on the association between the Q192R gene variation and CVD are controversial since not all populations show an association between this polymorphism and heart disease risk, as has been described previously [11].

The aim of this study was to identify the PON1 Q192R polymorphism and the OP exposure in adult coffee farmers of central Colombia and evaluate their association with the CVD risk through the respective analysis of the PON1 Q192R genotype, BChE activity, and lipid profile.

## 2. Materials and Methods

### 2.1. Study Population

A cross-sectional study was conducted on subjects from a coffee growing area of the Department of Caldas, Colombia, members of the Cooperativa de Caficultores de Manizales. A total of 234 workers were selected to participate based on their coffee harvesting activity and potential for exposure to pesticides; of these, 205 agreed to participate in the study. The study population was recruited from January 2014 through December 2015. Data on demographics, medical history, lifestyle, and occupational characteristics were collected via standardized questionnaires by trained interviewers. Informed consent was obtained from all individual participants included in the study. All procedures performed in studies involving human participants were in accordance with the ethical standards of the Universidad Católica de Manizales Institutional Research Committee and with the 1964 Helsinki Declaration and its later amendments or comparable ethical standards.

### 2.2. Cardiovascular Risk

The expected risk to have a heart attack or stroke in the next 10 years was used as the estimate of cardiovascular risk of an individual. Atherosclerotic cardiovascular disease (ASCVD) is defined as coronary death or nonfatal myocardial infarction, or fatal or nonfatal stroke. We used the 10-year ASCVD Risk Calculator (http://tools.acc.org/ASCVD-Risk-Estimator/) based on the algorithm published in the 2013 American College of Cardiology/American Heart Association Guideline on the Assessment of Cardiovascular Risk [12]. We selected the 10-year ASCVD risk threshold of ≥7.5% as a clinically relevant risk threshold, as described in the previous document and elsewhere [13].

### 2.3. Laboratory Procedures

Fasting blood samples were obtained by venipuncture and collected into glass tubes for serum lipid determinations. Levels of cholesterol and triglycerides were determined by enzymatic methods with commercially available kits (GTLab, Rosario, Argentina). Serum BChE was measured at 25°C with an enzyme kinetic assay using butyrylthiocholine as substrate (SpinReact, Girona, Spain). Genomic DNA was isolated from whole blood cells using the Isolate II Genomic DNA Kit (Bioline, Taunton, MA) according to the manufacturer's instructions.

### 2.4. PON1 Genotyping

The Q192R (rs662) sequence variant of the PON1 gene was determined by real-time PCR with a Taqman-based allele discrimination assay (Termo Fisher Scientific, Waltham, MA) using a LightCycler Nano thermocycler (Roche Diagnostics, Indianapolis, IN).

### 2.5. Statistical Analysis

Statistical analyses were performed with the SPSS statistical package version 15 (SPSS Inc., Chicago, IL). Assumption of normal distribution for continuous variables was tested by Kolmogorov-Smirnov test of goodness of fit. Continuous data are presented as mean ± standard deviation (SD), and one-way analysis of variance (ANOVA) test was used for multiple group comparison. Non-normally distributed data was summarized as median and range. The Pearson correlation coefficient was used to test the association between scale variables when linearity and constant variance were observed. The Spearman correlation coefficient was used to test the degree of association between ordinal variables. Categorical variables are described as frequencies and percentages and comparison between groups was done using the Chi-square test. PON1 Q192R allele frequencies were estimated by gene counting methods and Chi-square goodness of fit test was used to assess whether allele frequencies deviate from Hardy-Weinberg equilibrium. Q192R allele frequencies of the variant (R) and common (Q) allele between outcome events were compared using unrestricted and dominant (variant allele carriers versus homozygotes for the common allele) and recessive (homozygotes for the variant allele versus all others) genetic models [14]. Odds ratios (OR) were calculated as estimates of relative risk for outcome events and 95% confidence intervals (CI) obtained by logistic regression. Two-sided tests were used to test all the null hypothesis and *p* value ≤ 0.05 was considered statistically significant.

## 3. Results and Discussion

### 3.1. Results

Table 1 shows the demographic characteristics and clinical parameters of subjects involved in coffee farming or agricultural-related labor activities. The cardiovascular risk factors of hypercholesterolemia, hypertension, and the 10-year ASCVD risk were present in 62%, 33%, and 22% of the population of study, respectively. Subjects with simultaneous risk factors of hypertension and hypercholesterolemia represented the 17.6% of the population of study, and 4.4% of the population combined the three CDV risk factors analyzed (i.e., hypertension, hypercholesterolemia, and 10-year ASCVD risk factors) (Table 2).

A positive significant correlation was found between total cholesterol and BChE activity (*r* = 0.314; *p* < 0.01). Although statistical analysis showed nonsignificant associations, weak positive correlations were observed between blood pressure and total cholesterol, BChE, or the 10-year ASCVD risk factor on the subjects of study (Table 3).

PON1 Q192R genotypes and alleles distribution are presented in Table 4. The genotypic distribution was in Hardy-Weinberg equilibrium (Chi-square = 1.36; *p* = 0.243). The frequency of the PON1 192R allele (C allele that results in the amino acid arginine, R) present in subjects from the present study (40%) was similar in range to those reported in Phase 3 of the 1000 Genomes project for the allele frequency of a Colombian population of Medellín (42%) and populations of Asian and Indo-Asian origin (38–43%) [15].

The clinical and lipid profile and BChE activities as a function of the PON1 Q192R genotype are shown in Table 4. The Chi-square analysis revealed a statistically significant difference among blood pressure and the PON1 Q192R sequence variant; although the statistical analysis showed a marginal association between hypertension and the PON1 QQ genotype (Phi coefficient = 0.186), there was a significant difference (*p* = 0.029) between the frequencies of the PON1 QQ genotype of the hypertensive subjects (48%) and those with normal blood pressure levels (34%) in the unrestricted genetic model. A significant association was also observed under the dominant (*p* = 0.038) and recessive (*p* = 0.021) genetic models. No other significant differences were observed between any clinical or biochemical parameters and the PON1 polymorphism.

### 3.2. Discussion

Detection of subjects at risk constitutes a crucial issue when several health risk factors are present within a population. Environmental factors like chronic exposure to moderate levels of pesticides and health risk factors like hypertension and hyperlipidemia are prevalent among coffee harvesters of central Colombia [1, 16]. PON1 activity is a common molecular mechanism of protection to OP pesticide intoxication and CVD risk, and the gene PON1 Q192R polymorphism modulates the activity of the enzyme. Therefore, the present study aimed to elucidate the effect of PON1 Q192R sequence variant on OP intoxication and CVD risk of coffee harvesters in Colombia.

Women and men participating in the project are involved in different tasks on the coffee farming activity, and, hence, exposure to OPs is not similar for all the participants. Our results on the plasmatic BChE activity were all within the range of the reported reference values in a Colombian population (4,796.3–11,180.4 U/L, i.e., 79.94–186.34 *μ*kat/L) [17]. The BChE test is selected as biomonitor for low levels of exposure to OP since the onset of BChE enzyme inhibition by the OP exposure is earlier and the duration of depression (1–3 months) is longer than that for AChE [18]; also, although blood AChE and plasma BChE levels are used as surrogate activities of AChE in neural tissue, the BChE test assay can be measured faster and easier than the AChE assay [2]. However, the wide range of normal BChE activity originated by large interindividual and intraindividual differences makes identification of BChE inhibition by OP exposure hard to detect [18]. A drawback of our study was the impossibility to repeat the BChE test on the same individual to evaluate the percentage of change after the agricultural labor or a given period of time, since it has been proposed that a biological limit of 50% reduction on BChE activity is better indicator of OP exposure risk than the reference to a “normal” value [19, 20]. However, despite not having intraindividual BChE values in our study, the mean value of BChE activity (157.47 *μ*kat/L) was 15.5% below the upper limit of the reference range previously described; therefore we assume the subjects were not being exposed or recently exposed to OP or they were not at risk of OP toxicity at the time of the analysis.

In our study, hypertension was more frequent in women (54%) than men (29%) (data not shown), a difference higher in women than that reported for the Colombian population where the prevalence in 2014 was 31% in women and 29% in men [21]. A follow-up on this gender-related health risk factor might be needed to understand and confirm the observed result. Presence of hypertension and hypercholesterolemia in the overall population of our study of 33% and 62%, respectively, is higher for hypertension and identical for hypercholesterolemia to that observed by Gonzalez et al., [16] in a related coffee growing population of Colombia (26% and 62%, accordingly). In addition, as described in the previous study when comparing their results to the study of 1999 on chronic disease risk factors in Colombia (ENFREC II), “no important advances were found regarding a reduction of the prevalence of risk factors,” which unfortunately continues to be affirmed in this work.

The positive significant correlation observed in our study between total cholesterol levels and BChE activity supports the potential use of the BChE test to assess CVD risk along with classical CVD risk biomarkers. Several authors have reported the association of BChE activity with total cholesterol levels and cardiovascular risk. Wilson et al. [22], in a study of 5,345 subjects, described a multivariable-adjusted attributable risk percent of 27% in men and 34% in woman of total cholesterol levels associated with coronary heart disease. Vallianou et al. [23] demonstrated the positive association of BChE activity with BMI, LDL-cholesterol, total cholesterol, and triglycerides, stating a hypothesis for BChE as a novel marker of atherosclerotic disease. In addition, previous investigators have found significant relationships between BChE activity and obesity, metabolic syndrome, and diabetes risk [24–26]. It has been described that plasma BChE clusters with a range of characteristics, like body mass index, apolipoprotein, insulin, liver enzymes, and blood pressure [27], which might be partially determined by the rate of BChE production in the liver occurring in a coupling fashion with proteins synthesized under adverse metabolic environments [28].

PON1 is an antioxidant protein mainly synthetized in the liver that has direct interactions with proteins associated with cardiovascular regulation like apolipoproteins A1, A2, C3, E, and J and albumin, cholesteryl ester transfer protein, and BChE [29]. The activity of PON1 is under genetic regulation and varies widely between individuals and populations [30]. The expression of the protein depends mainly on two polymorphisms located in the coding region of the gene, the glutamine 192 arginine (Q192R) and leucine 55 methionine (L55M) PON1 polymorphisms [10]. Many studies have examined the link between the coding PON1 polymorphisms and CVD risk and, particularly, the association of the PON1 Q192R gene variant with vascular diseases [14, 31–33]. Therefore, we analyze the PON1 Q192R gene variant in the coffee harvesters of the central region of Colombia. We found allele frequencies of PON1 Q192R gene variant of 0.60 for 192Q allele and 0.40 for 192R allele, which are similar to the allele frequency of a Colombian population of Medellín reported in the 1000 Genomes project and closer to those of indigenous American [34] and Indo-Asian population and different from those of Caucasians and African subjects [15, 35]. Geographically, the Coffee Belt region is in the Cordillera Central of the Colombian Andes. The ancestry analysis of this region has identified a high European and Native American genetic contribution and a low value of African ancestry [36]. The population of our study self-reportedly identified as mestizo and white individuals and a minority of Afro-descendant ethnic origin. Based on the stratified population in terms of ancestry and the observed PON1 allele frequency of our study, we consider that a close admixture estimation including multilocus single gene variants datasets of the individual ancestries should be taken into account for future studies. The 192R allele gene variant has been associated with a less active isoform towards lipoprotein oxidation than the Q allele, which results in a higher risk of vascular disease [37, 38]. Diverse meta-analysis reports have found the 192R gene variant associated with a small increase in the risk of ischemic stroke [14] and coronary heart disease [39]. However, other studies and meta-analysis reports have found no conclusive association on PON1 polymorphism and CVD [40–42] or have reported the 192Q allele as the allele of CDV risk [33, 43, 44]. Several authors have reviewed the conflictive results and coincided with the importance of analyzing the PON1 status levels and PON1 haplotypes and the exogenous factors that concur with and modify the PON1 activity levels and therefore affect importantly the relation with CVD risk [29, 45, 46]. In the present work, we found an association of the PON1 192Q allele with hypertension in the unrestricted, dominant, and recessive genetic models evaluated; however, we did not find association with other clinical characteristics or the 10-year ASCVD risk in the Colombian coffee harvester population of our study. The influence of the PON1 192Q allele on disease susceptibility has been documented previously. Shahmohamadnejad et al. [47] observed the lower arylesterase activity of rheumatic patients having the 192Q allele and reported it as a contributor to the severity of the disease compared with subjects with the 192RR genotype. Also, in a structural model, it was demonstrated that the PON1 192Q protein binds HDL with a 3-fold lower affinity than the R isozyme and consequently exhibits significantly reduced stability, lipolactonase activity, and macrophage cholesterol efflux [48], a mechanism considered in the increased susceptibility of PON1 192Q allele to atherosclerosis. The study of factors like diet and pharmacological agents that influence the regulation of PON1 activity must be considered to increase our understanding of the interaction of gene environment and cardiovascular risk in the population of our study.

## 4. Conclusion

In the present work, we detected a high frequency of hypertension and hypercholesterolemia in the coffee harvester population of central Colombia, which underlines the need of public health interventions to reduce the risk factors associated with CVD. Our findings support the association of the PON1 192Q allele with hypertension, which remarks the idea to consider the impact of the PON1 activity and the role of its genetic, nutritional, and environmental modulators to favor a better understanding of the involvement of PON1 as a health protective factor on the CVD risk.

## Figures and Tables

**Table 1:** Demographic characteristics and laboratory profile of the study population (*n* = 205).

Characteristic	Value
*Sex*	
Male	168 (82)
Female	37 (18)
*Age* (years)	52 (27–84)
*Body mass index* (kg/m^2^)	24.0 ± 3.6
*Exercise*	129 (63)
*Current smoking*	27 (13)
*Family history of early hearth disease*	40 (20)

*Self-reported ethnicity*	
Mestizo	125 (61)
White	72 (35)
Afro-descendant	8 (4)

*Butyrylcholinesterase* (*μ*kat/L)	157.47 ± 32.8

*HDL cholesterol* (mg/dL)	56.3 ± 16.4
*Total cholesterol* (mg/dL)	218.4 ± 47.2
*Total cholesterol*	
Desirable (<200 mg/dL)	77 (38)
Borderline high (200–239 mg/dL)	66 (32)
High (≥240 mg/dL)	62 (30)
*Total cholesterol*	
Normal (<200 mg/dL)	*77 (38)*
Hypercholesterolemia (≥200 mg/dL)	*128 (62)*

*Blood pressure*	
Normal	33 (16)
(<120 mm Hg systolic or <80 mm Hg diastolic)	
Prehypertension	104 (51)
(120–139 mm Hg systolic or 80–89 mm Hg diastolic)	
Stage I hypertension	52 (25)
(140–159 mm Hg systolic or 90–99 mm Hg diastolic)	
Stage II hypertension	16 (8)
(≥160 mm Hg systolic or >100 mm Hg diastolic)	
*Blood pressure*	
Normal (<140 mm Hg systolic or <90 mm Hg diastolic)	*137 (67)*
Hypertension (≥140 mm Hg systolic or >90 mm Hg diastolic)	*68 (33)*

*10-year ASCVD risk*	
Low (<7.5%)	*130 (78)*
High (≥7.5%)	37 (22)

Data are presented as *n* (%), median (min–max), or mean ± SD.

**Table 2:** Association between CVD risk factors in coffee harvesters.

		*Blood pressure*		
		Normal	Hypertension	*p*

*10-year ASCVD risk*				
	*Total cholesterol*			
Low	Normal	40 (19.5)	12 (5.9)	0.145
	Hypercholesterolemia	67 (32.7)	36 (17.6)	
	*Total cholesterol*			
High	Normal	14 (6.8)	11 (5.4)	0.773
	Hypercholesterolemia	16 (7.8)	9 (4.4)	

Data are presented as *n* (%). *p* values were obtained by Chi-square test.

**Table 3:** Overall correlations among markers of organophosphate exposure and CVD risk.

	Butyrylcholinesterase	Total cholesterol	Blood pressure
Butyrylcholinesterase^a^	-		
Total cholesterol^a^	0.314^*∗∗*^	-	
Blood pressure^b^	0.135	0.090	-
10-year ASCVD risk^b^	−0.116	−0.053	0.063

^a^Pearson correlation; ^b^Spearman Rho correlation. Statistically significant ^*∗∗*^*p* < 0.01.

**Table 4:** Markers of organophosphate exposure and CVD risk according to the PON1 Q192R genotype.

Allele	Q 248 (60)		R 162 (40)		Dominant model			Recessive model		
Genotype	QQ 79 (38)	QR 90 (44)	RR 36 (18)							
Parameters	QQ	QR	RR	*p*	QQ	QR + RR	*p*	QQ + QR	RR	*p*
*Butyrylcholinesterase* ^a^ (*μ*kat/L)	155.06 ± 32.07	160.26 ± 30.63	155.80 ± 39.46	0.559	155.06 ± 32.07	158.98 ± 33.39	0.406	157.83 ± 31.32	155.80 ± 39.46	0.736

*HDL cholesterol* ^a^ (mg/dL)	56.5 ± 17.7	55.9 ± 15.6	57.1 ± 15.8	0.931	56.5 ± 17.7	56.2 ± 15.6	0.898	56.2 ± 16.6	57.1 ± 15.8	0.776

*Total cholesterol* ^a^ (mg/dL)	221.6 ± 41.0	211.8 ± 48.4	228.0 ± 55.4	0.166	221.6 ± 41.0	216.5 ± 50.8	0.452	216.4 ± 45.2	228.0 ± 55.4	0.180

*Total cholesterol* ^b^										
Normal	24 (31)	40 (52)	13 (17)	0.166	24 (31)	53 (69)	0.093	64 (83)	13 (17)	0.843
Hypercholesterolemia	55 (43)	50 (39)	23 (18)		55 (43)	73 (57)		105 (82)	23 (18)	
					1.664 [0.917–3.019]			0.927 [0.439–1.959]		

*Blood pressure* ^b^										
Normal	46 (34)	61 (44)	30 (22)	0.029^*∗*^	46 (34)	91 (66)	0.038^*∗*^	107 (78)	30 (22)	0.021^*∗*^
Hypertension	33 (48)	29 (43)	6 (9)		33 (48)	35 (52)		62 (91)	6 (9)	
					1.865 [1.031–3.376]			2.897 [1.142–7.348]		

*10-year ASCVD risk* ^b^										
Low	58 (37)	66 (43)	31 (20)	0.271	58 (37)	97 (63)	0.563	124 (80)	31 (20)	0.106
High	21 (42)	24 (48)	5 (10)		21 (42)	29 (58)		45 (90)	5 (10)	
					1.211 [0.633–2.318]			2.250 [0.824–6.142]		

Data are expressed as *n* (%), mean ± SD, and odds ratio [95% CI]. *p* values were obtained by ^a^one-way ANOVA and ^b^Chi-square test. ^*∗*^Statistically significant (*p* < 0.05).
